# LncRNA CBR3-AS1 potentiates Wnt/β-catenin signaling to regulate lung adenocarcinoma cells proliferation, migration and invasion

**DOI:** 10.1186/s12935-020-01685-y

**Published:** 2021-01-09

**Authors:** Min Hou, Nannan Wu, Lili Yao

**Affiliations:** Clinical Laboratory, Tianjin Chest Hospital, No. 261, South Taierzhuang Road, Tianjin, 300222 China

## Abstract

**Background:**

Long non-coding RNAs (lncRNAs) are pervasively transcribed in genome and emerging as a new player in tumorigenesis due to their functions in transcriptional, posttranscriptional and epigenetic mechanisms of gene regulation. As the most frequent malignancy and the foremost source of cancer mortality, lung cancer is a heterogeneous disorder. The most common type of lung cancer is Non-small cell lung cancer (NSCLC), occupying 85% of the total cases, and the main subtypes of NSCLC include lung adenocarcinoma (LAD), large cell carcinoma (LCC), and lung squamous cell carcinoma (LSCC). Recently, numerous lncRNAs have been reported to be strongly linked to NSCLC. In the present study, we found that a new lncRNA CBR3-AS1 is highly expressed in lung cancer. In addition, we also examined the expression of lncRNA CBR3-AS1 in 60 of LADs, 40 of LCCs and 40 of LSCCs patient samples, finding that CBR3-AS1 was specificity highly expressed in LAD cancer tissues. Mechanically, we discovered that CBR3-AS1 could regulate the proliferation, migration and invasion of LAD cells through targeting Wnt/β-catenin signaling.

**Methods:**

Real-time PCR, RNA-pulldown, RIP, western blotting, lentivirus transfection, luciferase reporter assays, cell proliferation assays, colony formation assays, wound healing scratch assays and transwell assays were employed to examine the relationship between lncRNA CBR3-AS1 and its regulation of Wnt/β-catenin signaling in LAD cells.

**Results:**

LncRNA CBR3-AS1 is highly-expressed in LAD and cell lines. LncRNA CBR3-AS1 shows physical association with β-catenin. CBR3-AS1 could facilitate Wnt/β-catenin signaling activation thought promoting nuclear localization of β-catenin. CBR3-AS1 promotes LAD cell proliferation, migration and invasion by targeting Wnt/β-catenin signaling.

**Conclusion:**

It can be found that a new functional lncRNA CBR3-AS1 could promote nuclear localization of β-catenin so as to facilitate Wnt/β-catenin signaling activation and regulate the proliferation, migration and invasion of LAD cells.

## Introduction

Lung cancer is considered as one of the most common cancers globally, affecting both genders [1]. Two types of main lung cancer are small cell lung cancer (SCLC) and non-small cell lung cancer (NSCLC). SCLC accounts for 15–20% of people that diagnosed with lung cancer, and is more aggressive than NSCLC which can spread rapidly [2]. In addition, NSCLC is the most common type of lung cancer, which approximately accounts for 80–85% of people that diagnosed with lung cancer [3, 4]. NSCLC can be further divided into three subtypes, respectively, lung adenocarcinoma (LAD), large cell carcinoma (LCC) and lung squamous cell carcinoma (LSCC), based on its pathological characteristic. However, LAD accounts for approximately 40% of all lung cancer patients and there is an urgent requirement to understand the mechanisms of cancer progression in LAD and find out useful biomarkers to predict prognosis [5]. Recently, target therapies, for instance, inhibiting the epidermal growth factor receptor (EGFR) through tyrosine kinase inhibitors (TKIs) [6] and immune checkpoint inhibitors, have been triumphantly used in clinical diagnose and treatments despite that traditional therapeutic strategies have been tremendously improved while the survival rate of lung cancer combined remains extremely low [2]. Such poor outcome should at least partially attribute to the poor understanding about the pathogenesis of lung cancer, especially non-small cell lung cancer. As a result, it seems more important to find more functional biomarkers and therapeutic targets.

Recently, high-throughput transcriptome analysis shows that nearly 75 percent of genome can be transcribed into RNAs and a few of them translate to proteins with others as noncoding RNAs (ncRNAs) [7]. According to the length, ncRNAs can be divided into two types, RNAs shorter than 200 bp, such as microRNAs (miRNAs), small interfering RNA (siRNAs), PIWI-interacting RNAs (piRNAs), housekeeping ncRNAs, small nuclear RNAs (snRNAs), and small nucleolar RNAs (snoRNAs) and RNAs longer than 200 bp containing the long non-coding RNAs (lncRNAs) [8, 9]. Long non-coding RNAs (lncRNAs) are pervasively transcribed in genome, emerging as a new player in tumorigenesis which attributes to its functions in transcriptional, posttranscriptional and epigenetic mechanisms of gene regulation [9, 10]. LncRNAs could interact with RNA, DNA, and/or proteins to regulate chromatin modifications or structure, transcription, pre-mRNA splicing and translation, or as scaffolds for protein complex assembly [11]. Consequently, lncRNAs could participate in the regulation of physiological and pathological processes, such as cell proliferation or differentiation, tumorigenesis, drug resistance or stem cell reprogramming [12–14].

In recent years, an increasing number of evidences demonstrated that the lncRNAs exert important roles in a variety of diseases, especially in cancer. In NSCLC, there are a variety of lncRNAs with high expression levels as previous reports and some of them play significant roles. For example, CAR10 can bind and stabilize TFY-box-binding protein 1 (YBX-1) to up-regulate the expression of EGFR and proliferation of lung cancer cells [15]. In addition, some functional lncRNAs which have been extensively investigated possess certain roles in the pathogenesis of NSCLC, such as, H19, HOTAIR, MALAT1, PVT1 and NKX2-AS1 [16–20]. Hence, funding more functional lncRNAs would provide prominent contributions to the diagnosis and treatment of NSCLC as well as LAD.

Wnt/β-catenin signaling pathway is an important molecular cascade in regulating embryonic development and adult homeostasis [21–23]. Moreover, its dysregulation has been implicated in human malignancies including colorectal cancer, breast cancer, prostate cancer and non-small cell lung cancer [24–26]. In canonical Wnt signaling pathway, as the central protein, β-catenin nuclear transport controls the activation of them and promotes the expression of Wnt target genes, such as LGR5, c-Myc, Cyclin D1, CD44 and MMP-7 [27–31]. Wnt signaling pathway exerts a significant influence in NSCLC cell lines, impairs the Wnt signaling could inhibit the proliferation [32] and xenograft growth, reduce cell migration and invasion of NSCLC cells [33, 34] as well as induce more differentiated phenotypes.

## Results

### LncRNA CBR3-AS1 is highly-expressed in LAD and cell lines

**Fig. 1:**
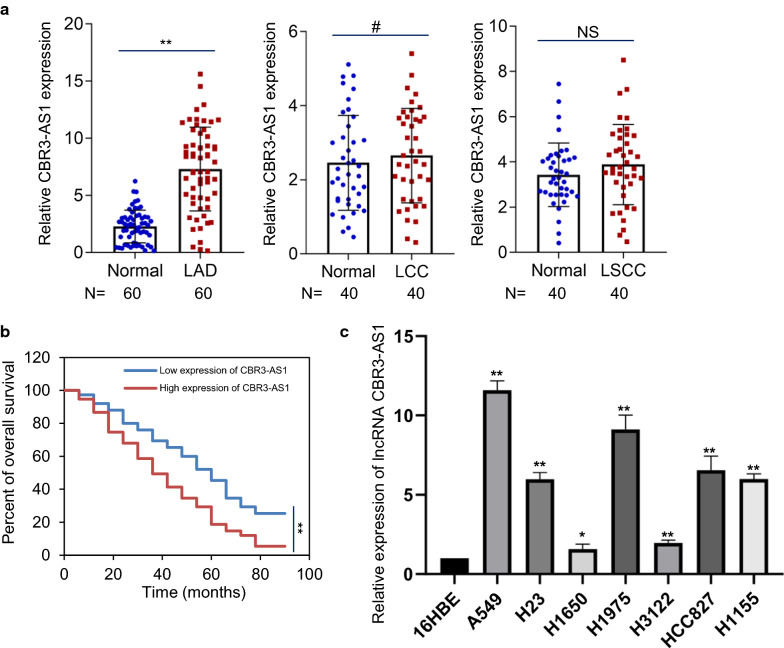
LncRNA CBR3-AS1 is highly-expressed in LAD and cell lines. **a** qRT-PCR analysis the expression of lncRNA CBR3-AS1in LAD, LCC or LSCC patient tissues compared with its adjacent non-cancerous tissues. LAD: n = 60; LCC: n = 40; LSCC: n = 40. **P < 0.01, ^#^*P* > 0.05, one-way analysis of variance (ANOVA). **b** LAD patients with lncRNA CBR3-AS1 high-expression have shorter overall survival compared to patients with lncRNA CBR3-AS1 low-expression. n = 75, **P < 0.01, two-way ANOVA. **c** Levels of lncRNA CBR3-AS1 are elevated in LAD cell lines compared with human bronchial epithelial cell line. 0.01 < *P < 0.05, **P < 0.01, one-way ANOVA

Long non-coding RNAs (lncRNAs) govern fundamental biochemical and cellular processes. To assess more important functional lncRNAs in non-small cell lung cancers (NSCLC), we examined the lncRNA-disease related database LncRNADisease v2.0 (http://www.rnanut.net/lncrnadisease/index.php/home/search), which was integrated comprehensive experimentally support and predicted ncRNA-disease associations curated from manual literatures and other resources [13, 35, 36], finding that a latent functional lncRNA, CBR3-AS1, is highly-expressed in NSCLC. To further validate the obtained results in database, we detected the expression of lncRNA CBR3-AS1 in three subtypes of NSCLC, 60 LAD, 40 LCC and 40 LSCC patient tumor tissues compared with its normal specimens. The results could be found in Fig. 1a and Additional file 1: Figure S1A. lncRNA CBR3-AS1 is highly-expressed in LAD tissues, and lncRNA CBR3-AS1shows positive correlation with poor prognosis of LAD patients (Fig. 1b). To further dissect these phenomena, we detected the expression level of lncRNA CBR3-AS1 in LAD cell lines. The results indicated that lncRNA CBR3-AS1 is highly expressed in LAD cell lines (Fig. 1c). Collectively, these results prove that CBR3-AS1 was highly expressed in LAD tissues and cell lines, indicating that lncRNA CBR3-AS1 may play an important role in the occurrence of non-small cell lung cancer.

### LncRNA CBR3-AS1 is physically associated with β-catenin

**Fig. 2:**
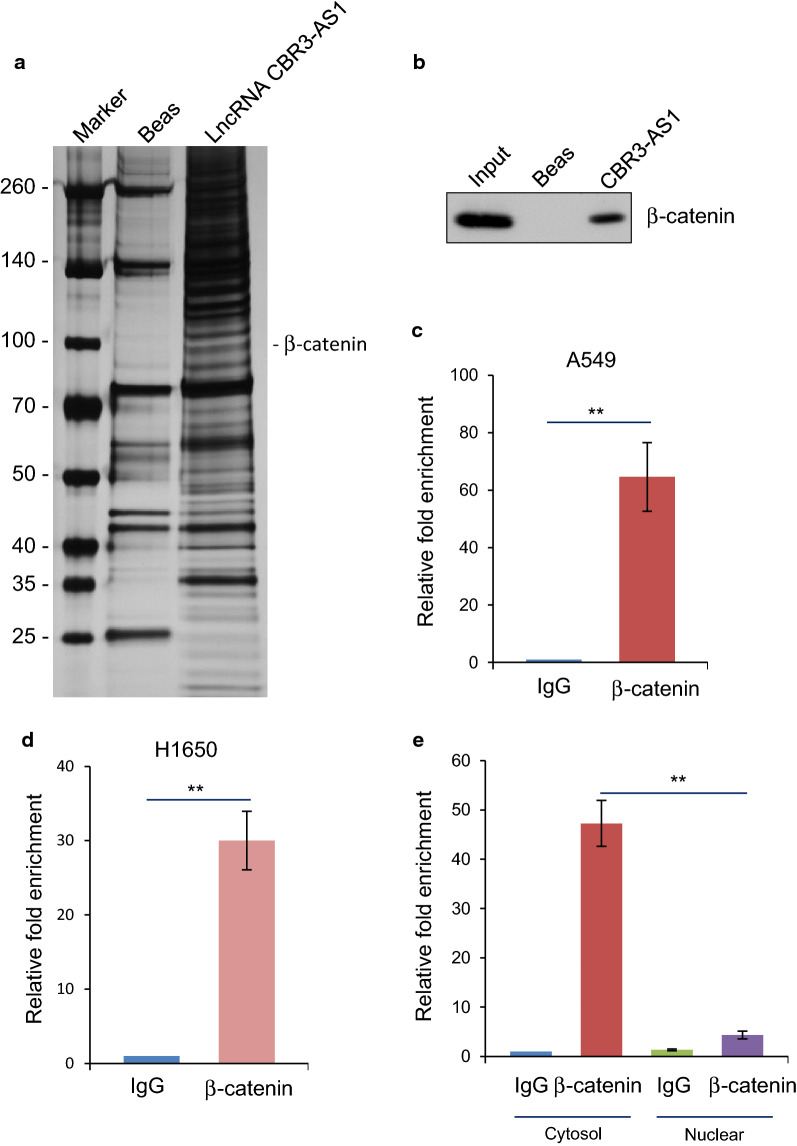
LncRNA CBR3-AS1 is physically associated with β-catenin. **a** LncRNA CBR3-AS1 RNA pull-down assay and mass spectrometry identified CBR3-AS1-interacting proteins and visualized by silver staining on SDS-PAGE. Detailed results from the mass spectrometric analysis are provided as Additional file 3: Table S2. **b** LncRNA CBR3-AS1 RNA pull-down assay was examined by western blotting with antibody against β-catenin. **c** RNA immunoprecipitation (RIP) assays with anti-β-catenin antibody and followed by qRT-PCR assays in A549 cells. **P < 0.01, one-way ANOVA. **d** RNA immunoprecipitation (RIP) assays with anti-β-catenin antibody and followed by qRT-PCR assays in H1650 cells. **P < 0.01, one-way ANOVA. **e** RNA immunoprecipitation (RIP) assays with anti-β-catenin antibody against different cellular components and followed by qRT-PCR assays in A549 cells. **P < 0.01, one-way ANOVA

In order to better understand the mechanistic role of lncRNA CBR3-AS1 in the progenesis of non-small cell lung cancers, we employed RNA pull-down assay using an in vitro transcribed CBR3-AS1 and incubated with cell lysates from A549 cells in combination with mass spectrometry (MS) analysis, aiming to detect potential CBR3-AS1 associated proteins. This analysis identified β-catenin as a possible CBR3-AS1-interacting protein (Fig. 2a). This interaction was validated by in vitro CBR3-AS1 RNA pull-down assay and followed by western blotting assays against with anti-β-catenin antibody in A549 cells (Fig. 2b). Simultaneously, we employed an RNA immunoprecipitation (RIP) assays with anti-β-catenin antibody and followed by qRT-PCR assays (Fig. 2c). The above results potentiated the interaction of lncRNA CBR3-AS1 with β-catenin (Additional file 2).

To confirm the interaction between lncRNA CBR3-AS1 and β-catenin, we conducted another RNA pull-down assay and RIP assays in another LAD cell line, H1650 cells, indicating that the interaction between lncRNA CBR3-AS1 and β-catenin is prevalent in LAD cells (Fig. 2d). In order to further characterize the interaction between lncRNA CBR3-AS1 and β-catenin in different cellular components, we employed RIP assay to analyze the point interact station. The result indicated that β-catenin could interact with CBR3-AS1 in cytosolic in A549 cells (Fig. 2e). In summary, these results suggest that a potential intracytoplasmic function of CBR3-AS1 with β-catenin.

### CBR3-AS1 facilitates Wnt/β-catenin signaling activation by promoting nuclear location of β-catenin in LAD cell lines

**Fig. 3:**
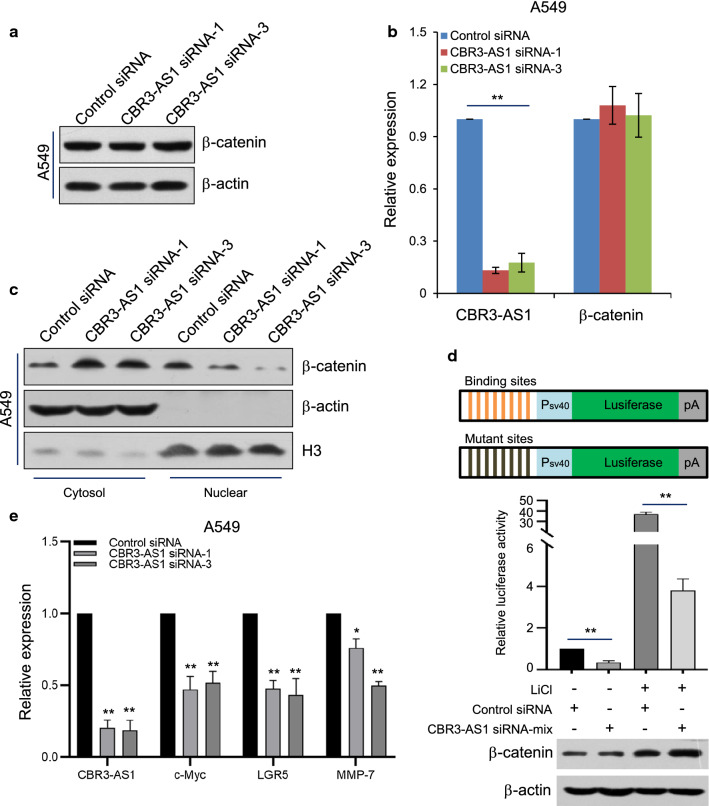
CBR3-AS1 facilitates Wnt/β-catenin signaling activation by promoting nuclear location of β-catenin in LAD cell lines. **a** A549 cells were transfected with control siRNA or CBR3-AS1 siRNA and the expression of β-catenin was examined by western blotting with antibody against β-catenin. **b** A549 cells were transfected with control siRNA or CBR3-AS1 siRNA and the expression of CBR3-AS1 and β-catenin was examined by qRT-PCR assays. Each bar represents the mean ± SD for biological triplicate experiments. **P < 0.01, one-way ANOVA. **c** A549 cells were transfected with control siRNA or CBR3-AS1 siRNA and the cellular components were extracted followed by western blotting with antibody against β-catenin. **d** TopFlash/FopFlash-luciferase reporter constructs are as shown (upper panel). A549cells were co-transfected with control siRNA or CBR3-AS1siRNA together with renilla and pGL3-TopFlash orpGL3-FopFlash. These cells were cultured in the absence or presence of 25 mM LiCl for 4 h before measuring luciferase activity. The relative luciferase activity was normalized with values of renilla luciferase and pGL3-FopFlash luciferase activity. Western blotting against with indicated antibodies. Each bar represents the mean ± SD for biological triplicate experiments. **P < 0.01, one-way ANOVA. **e** A549 cells were transfected with control siRNA or CBR3-AS1 siRNA and the expression of CBR3-AS1, c-Myc, LGR5 and MMP-7 was examined by qRT-PCR assays. Each bar represents the mean ± SD for biological triplicate experiments. **P < 0.01, one-way ANOVA

To further characterize the function of CBR3-AS1, we transfected the control siRNA or CBR3-AS1 siRNA into A549 cells and also detected the expression level of β-catenin. The results suggested that knocking down CBR3-AS1 could not change the protein level of β-catenin (Fig. 3a). Furthermore, knockdown of CBR3-AS1exerts no effect on β-catenin mRNA levels (Fig. 3b). However, in order to better understand the mechanism of CBR3-AS1, we analyzed the expression of β-catenin in different cellular components with transfected with control or CBR3-AS1 siRNA. The results demonstrated that the knockdown of CBR3-AS1 could increase the cytoplasmic β-catenin levels and reduce the nuclear β-catenin levels (Fig. 3c). According to the obtained results, CBR3-AS1 could increase nuclear location of β-catenin in A549 cells.

Admittedly, β-catenin is a central protein of canonical Wnt signaling pathway and its nuclear transport controls the activation of canonical Wnt signaling pathway [37, 38]. To assess the effect of CBR3-AS1 on Wnt signaling pathway, pGL3-TopFlash reporter containing eight tandem repeats of TCF/LEF binding elements or its counterpart pGL3-FopFlash reporter containing mutated TCF/LEF binding elements (Fig. 3d) were co-transfected into A549 cells together with CBR3-AS1 siRNA and renilla luciferase vector. Subsequently, A549 cells were cultured in the absence or presence of LiCl, inhibiting GSK3, which can thus stabilize the level of β-catenin and then increase its nuclear transport and activate the canonical Wnt pathway. The result of reporter assays showed that CBR3-AS1 gene knockdown significantly reduced the TCF/LEF reporter activity in response to LiCl stimulation (Fig. 3d). To further characterize the effect of CBR3-AS1 on Wnt signaling pathway, A549 cell were transfected with control or CBR3-AS1 siRNA and then subjected to qRT-PCR or Western blotting assays so as to analyze the expression of Wnt target genes, such as, c-Myc, LGR5 and MMP-7 (Fig. 3e and Additional file 1: Figure S2A). The results suggested that CBR3-AS1 knockdown reduced the expression of Wnt target genes. Collectively, these obtained results demonstrate that CBR3-AS1 could facilitate canonical Wnt signaling pathway activation thought promoting the nuclear localization of β-catenin.

### CBR3-AS1 promotes LAD cell proliferation by targeting Wnt/β-catenin signaling

**Fig. 4:**
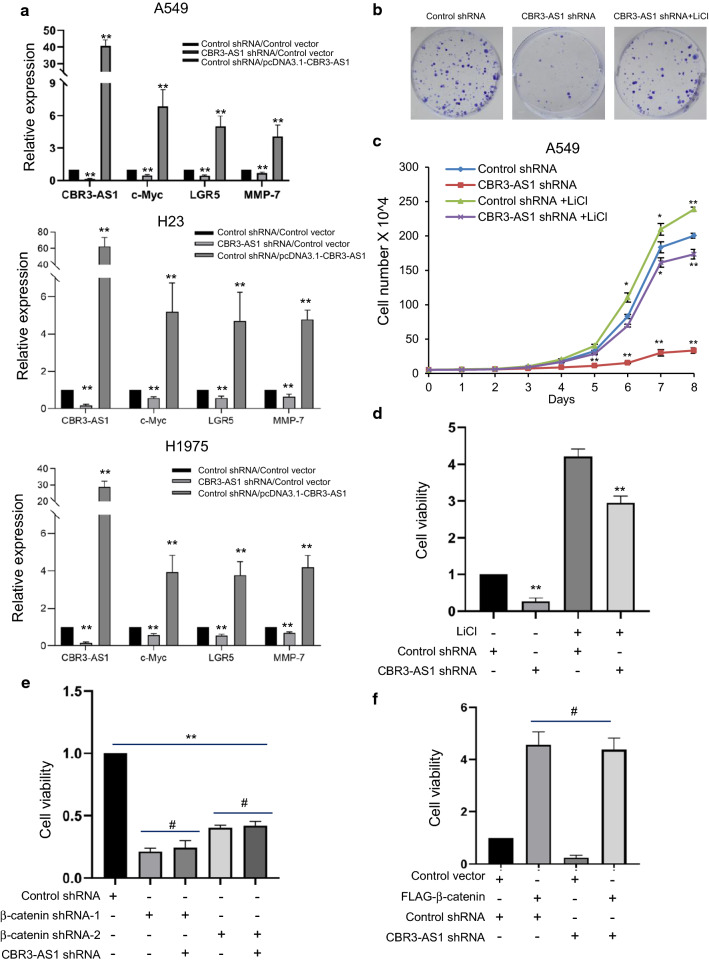
CBR3-AS1 promotes LAD cell proliferation by target Wnt/β-catenin signaling. **a** A549, H23 or H1975 cells were stably transfected with control shRNA or CBR3-AS1 siRNA or transfected with pcDNA3.1-CBR3-AS1 and examined the expression of CBR3-AS1, c-Myc, LGR5 and MMP-7 by qRT-PCR assays. Each bar represents the mean ± SD for biological triplicate experiments. **P < 0.01, one-way ANOVA. **b** Colony formation assays with A549 cells stably expressing control shRNA or CBR3-AS1 shRNA and cultured in the absence or presence of 25 mM LiCl for 2 weeks. **c** Growth viability assay with A549 cells stably expressing control shRNA or CBR3-AS1 shRNA and cultured in the absence or presence of 25 mM LiCl for 8 days. Each bar represents the mean ± SD for biological triplicate experiments. 0.01 < *P < 0.05, **P < 0.01, one-way ANOVA. **d** Cellular viability assay by CCK8 kit with A549 cells stably expressing control shRNA or CBR3-AS1 shRNA and cultured in the absence or presence of 25 mM LiCl. Each bar represents the mean ± SD for biological triplicate experiments. **P < 0.01, one-way ANOVA. **e** Cellular viability assay by CCK8 kit with A549 cells stably expressing control shRNA or β-catenin shRNA or co-transfected with β-catenin and CBR3-AS1 shRNAs. Each bar represents the mean ± SD for biological triplicate experiments. ^#^*P* > 0.05, **P < 0.01, one-way ANOVA. **f** Cellular viability assay by CCK8 kit with A549 cells stably expressing control shRNA or CBR3-AS1shRNA or co-transfected with FLAG-β-catenin and CBR3-AS1 shRNAs. Each bar represents the mean ± SD for biological triplicate experiments. ^#^*P* > 0.05, one-way ANOVA.

To further characterize the CBR3-AS1 regulated Wnt/β-catenin signaling pathway, we infect A549, H23 and H1975 cells with lentivirus containing shRNAs target CBR3-AS1. The expression of CBR3-AS1 was identified by qRT-PCR assays (Fig. 4a) and its knockdown decreased the expression of Wnt/β-catenin target genes, such as c-Myc, LGR5 and MMP-7, while the overexpression of CBR3-AS1 increased their expression. These results strengthen the conclusion that CBR3-AS1 promotes the activation of Wnt signaling pathway (Fig. 4a). Earlier studies reported that Wnt signaling controls proliferation, maturation and invasion in various types of cancer, including lung cancer. To understand the effect of CBR3-AS1 on the proliferation of LAD cells, we conducted the colony formation assays of A549 cells infected with CBR3-AS1 targeted shRNAs. The results demonstrated that knockdown of CBR3-AS1 severely impeded the colony formation of A549 cells (Fig. 4b and Additional file 1: Figure S3A). Moreover, CBR3-AS1 depletion-related effects on A549 cells could be alleviated by being conducted with LiCl for 2 weeks (Fig. 4b and Additional file 1: Figure S3A). To further consolidate the functional link between CBR3-AS1 and lung cancer cell colony formation ability, A549 or H1975 cells were cultured in the absence or presence of LiCl. In addition, cell number was counted for 8 days (Fig. 4c and Additional file 1: Figure S3B). Cell viability was examined with CCK-8 assays (Fig. 4d).

To further investigate whether the effect of CBR3-AS1 on LAD cells is through the nuclear transport of β-catenin, we infected with control or lentivirus carrying β-catenin shRNAs or co-infect CBR3-AS1 and β-catenin shRNAs into A549 cells and detect its cell viability by CCK-8 assays. The obtained result indicates that the effect of CBR3-AS1 knockdown on the survival of A549 cells will not be serious in the absence of β-catenin (Fig. 4e). Similarly, knocking down CBR3-AS1 could not prevent the increase of A549 cell survival induced by overexpression of β-catenin (Fig. 4f). Collectively, these results support the notion that CBR3-AS1 could regulate LAD cell proliferation though targeting Wnt/β-catenin signaling pathway.

### CBR3-AS1 regulates LAD cell migration and invasion through Wnt/β-catenin signaling

**Fig. 5:**
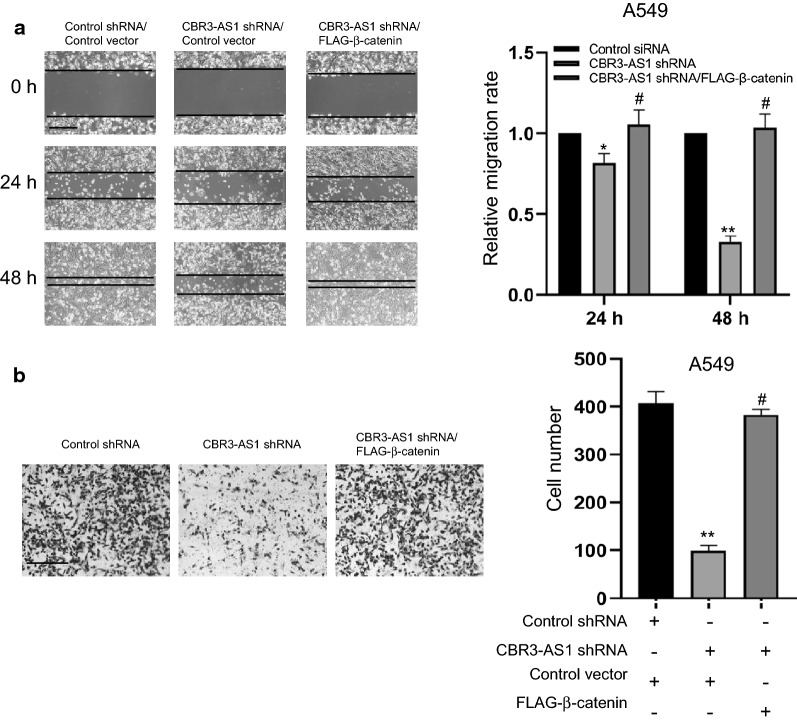
CBR3-AS1 regulates the LAD cell migration and invasion through Wnt/β-catenin signaling. **a** Wound healing scratch assays with A549 cells stably expressing control shRNA or CBR3-AS1 shRNA or co-transfected with FLAG-β-catenin. Each bar represents the mean ± SD for biological triplicate experiments. ^#^*P* > 0.05, 0.01 < *P < 0.05, **P < 0.01, one-way ANOVA. Scale bar, 50 μm. **b** A549 cells stably expressing control shRNA or CBR3-AS1 shRNA or co-transfected with FLAG-β-catenin followed by transwell invasion assays. Each bar represents the mean ± SD for biological triplicate experiments. ^#^*P* > 0.05, **P < 0.01, one-way ANOVA. Scale bar, 50 μm

It has been well documented that the Wnt/β-catenin signaling pathway is not only implicated in cell proliferation but also consociated with migration, invasion, and metastasis in various types of cancers [26]. In the present study, we discovered that CBR3-AS1 was upregulated in LAD cells and could regulate its proliferation. These results prompted us to explore the migration and invasion effects of CBR3-AS1 on LAD cells. Then, we assessed the migration ability through wound healing scratch assays under knockdown of CBR3-AS1 with shRNAs. Besides, the results show that the knockdown of CBR3-AS1 decreased migration ability of A549 and H1975 cells. Nevertheless, these could be rescued by the overexpression of β-catenin (Fig. 5a and Additional file 1: Figure S4A). In addition, the transwell invasion experiments further consolidated the function of CBR3-AS1 on LAD cell invasion and the result showed that CBR3-AS1 depletion inhibited the invasion of A549 cells and H1975, while these could also be rescued by the overexpression of β-catenin (Fig. 5b and Additional file 1: Figure S4B).

## Discussion

In the present study, we discovered a new highly-expressed functional lncRNA in LAD cells and tumor tissues of LAD patients. We identified that CBR3-AS1 could promote the nuclear transport of β-catenin to activate the canonical Wnt signaling pathway and induce the expression of its target genes, consequently regulating the proliferation, migration and invasion of LAD cells. In our results, RNA-pulldown and RIP assays identified the bona fide interaction between CBR3-AS1 and β-catenin. However, these interactions could not affect the expression of β-catenin when we knocking down CBR3-AS1 in total cells. Interestingly, knocking down CBR3-AS1 reduced the activation of Wnt signaling, suggesting that the reduction of CBR3-AS1 could probably change the amount of β-catenin in the cytoplasm or nucleus. Next, we analyzed the protein level of β-catenin in different cellular components under knocking down CBR3-AS1 through siRNAs, discovering the dynamic process of β-catenin transport to nuclear sites dependent on CBR3-AS1. This transport process promotes the activation of Wnt signaling pathway, increasing the expression of Wnt target genes, such as c-Myc [34], cyclin D1 [29], LGR5 [27], and MMP-7 [39] as well as regulate the proliferation, migration [40] and invasion [41] ability of NSCLC cells.

LncRNAs are relatively mature characterized class of ncRNAs, which has the function of facilitating or inhibiting the occurrence and development of tumors, undoubtedly including non-small cell lung cancer (NSCLC). On this basis, numerous functional lncRNAs have been discovered in NSCLC. For example, HOTAIR can interact with polycomb repressive complex 2 (PRC2) and lysine-specific demethylase 1 (LSD1) to regulate some genes, thereby regulating the migration and invasion of NSCLC cells [42]. The high expression of H19 is positively correlated with advanced tumor-node-metastasis (TNM) stage and tumor size [43]. The cancer-related region long non-coding RNA 5 (CARLo-5) promotes the EMT process of NSCLC cells [44, 45]. However, for all of these, finding more specific functional lncRNAs in NSCLC may represent more potential biomarker for the diagnosis and therapy target for NSCLC intervention.

In recent years, a large number of studies on RNA-binding proteins have been published, and RNA-binding protein family developed enormously and confusedly. RNA-binding proteins (RBPs) belong to a class of proteins that bind to the double or single stranded RNA in cells and participate in the formation of ribonucleoprotein complexes. While RBPs can be combined with sequence and/or structural motifs in RNA via modular combinations of structurally identified RNA-binding domains (RBDs) [46], such as RNA recognition motif (RRM) [47], hnRNP K homology domain (KH) [48] or DEAD box helicase domain [49]. However, in recent studies on determining the structures of large RNP machines, such as the spliceosome [50, 51] and ribosome [52, 53], finding the interaction between protein and RNA does not require canonical RBDs, and this unconventional RNA binding is more extensive than the previously anticipated. As the role protein of Wnt signaling pathway central, β-catenin interacted with the lncRNA CBR3-AS1 through some kinds of mechanisms such as RNA modifications and protein PTMs. Moreover, this part of questions needs to be further uncovered in our future studies.

## Methods

### Antibodies and reagents

The sources of antibodies against the following proteins were: β-actin (A1978, 1:10,000 for WB) from Sigma; β-catenin (51067-2-AP, 1:1000 for WB); Histone H3 (ab1791, 1:5000 for WB) from abcam.

### Plasmids

FLAG-β-catenin was carried by pLenti-Hygro vector. lncRNA CBR3-AS1 was carried by pcDNA3.1 vector. TopFlash DNA fragment coding eight tandem repeats of TCF response elements, and FopFlash DNA fragment as a negative control, carrying eight mutant TCF tandem repeats, were synthesized and introduced to pGL3-promoter vector upstream of the SV40 promoter to generate pGL3-TopFlash and pGL3-FopFlash respectively.

### Cell culture

A549, H23, H1650, H1975, H3122, HCC827, H1155 and 16HBE cells were got from the American Type Culture Collection (Manassas, VA) and cultured under the manufacturer’s instructions. All of the cultured cells were authenticated by examination of morphology and growth characteristics, and were confirmed to be mycoplasma-free.

### Western blotting

Cells were lysed by Laemmli sample buffer (161–0737, BioRad), and re-suspending in 5× SDS-PAGE loading buffer. The boiled protein samples were then subjected to sodium dodecyl sulfate polyacrylamide gel electrophoresis (SDS-PAGE) and transferred to nitrocellulose membrane incubated with appropriately primary antibodies and secondary antibodies (Additional file 2).

### RNA-pulldown and silver staining

The RNA pull-down assay was modified from previous studies [54]. Substrate RNAs were in vitro transcribed in a 200 µl reaction mix containing 1 μg of pcDNA3.1-CBR3-AS1 DNA detailed protocol as RiboMAX™ Large Scale RNA Production Systems-T7 manufacturer’s instructions (P1300, Promega). In addition, transcribed RNAs produced 3′ End Desthiobiotinylation using Pierce™ RNA 3′ End Desthiobiotinylation Kit (20163, Invitrogen) and incubated with A549 cell lysed by IP lysis buffer (87787, Invitrogen) for RNA-pulldown assays as Pierce™ Magnetic RNA–Protein Pull-Down Kit (20164, Invitrogen). Then, the eluents were collected and visualized on NuPAGE 4–12% Bis–Tris gel (NP0321BOX, Invitrogen) followed by silver staining with silver staining kit (24600, Invitrogen). The distinct protein bands were retrieved and analyzed by LC–MS/MS.

### Mass spectrometry (MS) analysis

lncRNA CBR3-AS1 interacted proteins performed LC–MS/MS analyzed by Thermo Finnigan LTQ linear ion trap mass spectrometer (Thermo Fisher Corporation, San Jose, CA) in line with a Thermo Finnigan Surveyor MS Pump Plus HPLC system. The mass spectrometry analysis was carried out in a data-dependent mode with an automatic switch between a full MS and an MS/MS scan in the obitrap. Peptide sequences were searched using trypsin specificity and allowing a maximum of two missed cleavages. Sequest was searched with a peptide tolerance of 3.0 Da and a fragment ion tolerance of 1.0 Da. The results of peptide sequences information of CBR3-AS1 interact proteins were offered in Additional file 3: Table S2.

### RNA immunoprecipitation (RIP)

For A549 cells, 1 × 10^7^ cells were harvested and crosslinked with 0.3% formaldehyde for 10 min at RT and quenched with 0.125 M glycine for 5 min. Nuclei were extracted and lysed in RIP Cross-Linked Lysis Buffer and product protocol supplied by Magna Nuclear RIP™ (Cross-Linked) Nuclear RNA-Binding Protein Immunoprecipitation Kit (17-10520, Millipore). In addition, RNAs were extracted with TRIzol and detected by qRT-PCR.

### RNA interference

In our studies, all siRNAs were transfect to cells using Lipofectamine RNAiMAX (Invitrogen) reagents following the manufacturer's recommendations. The final concentration of the siRNA molecules is 10 nM and cells were harvested 72 h. Control siRNA and the individual siRNAs against CBR3-AS1 were chemically synthesized by GenePharma. The short hairpin RNAs (shRNAs) against β-catenin or CBR3-AS1 were expressed as lentiviral, purchased from GenePharma, transfected into appropriate cells. The sequences of siRNAs and shRNAs are provided in Additional file 4: Table S3.

### qRT-PCR

Total cellular RNAs were isolated by TRIzol reagent (Invitrogen) and transcribed by the Reverse Transcription System (Roche). Quantitation of all gene transcripts was done by qPCR using a Power SYBR Green PCR Master Mix (Roche) and Q5 detection system (Thermo) with the expression of ACTB as the internal control. The primers used were listed in Additional file 4: Table S4.

### Colony formation assay

A549 cells stably expressing indicated genes or/and shRNAs were cultured in the absence or presence of LiCl for 2 weeks. After 2 weeks, the cells were washed and fixed with PBS and methyl alcohol respectively, then, stained with crystal violet (0.5% wt/vol). The number of colonies per well was counted.

### Cell viability assay

Indicated cells were cultured in appropriate media for 24 h in 96-well plates, 10 μl CCK-8 regents were added ahead of 2–4 h for test OD value of 450 nm. The detail protocols were supplied by Cell Counting Kit-8 (HY-K0301-500T, MedChem Express).

### Statistical analysis

Experimental data from biological triplicate experiments are presented with error bar as mean ± SD. Two-tailed unpaired Student’s t-test was used for comparing two groups of data. Analysis of variance (ANOVA) with Bonferroni’s correction was used to compare multiple groups of data. A P value of less than 0.05 was considered significant and higher than 0.05 was considered no specific differences. All of the statistical testing results were determined by SPSS 22.0 software diagrams were conducted using GraphPad prism 8.0. Before statistical analysis, variation within each group of data and the assumptions of the tests were checked.

## Supplementary Information

**Additional file 1: Figure S1.** LncRNA CBR3-AS1 is highly-expressed in LAD and cell lines. (A) The expression of lncRNA CBR3-AS1 in LAD (n = 483) or Normal (n = 347) and LSCC (n = 486) or Normal (n = 338) tissues from TCGA and GTEx database (http://gepia.cancer-pku.cn/detail.php?gene=CBR3-AS1). **P < 0.01, ^#^P > 0.05, one-way ANOVA. **Figure S2.** CBR3-AS1 facilitates Wnt/β-catenin signaling activation by promoting nuclear location of β-catenin in LAD cell lines. (A) A549 cells were transfected with control siRNA or CBR3-AS1 siRNA and the expression of c-Myc, LGR5 and MMP-7 was examined by western blotting assays. **Figure S3.** CBR3-AS1 promotes LAD Wnt/β-catenin signaling. (A) Quantitation colony number of colony formation assays with A549 cells stably expressing control shRNA or CBR3-AS1 shRNA and cultured in the absence or presence of 25 mM LiCl for 2 weeks. Each bar represents the mean ± SD for biological triplicate experiments. **P < 0.01, one-way ANOVA. (B) Growth viability assay with H1975 cells stably expressing control shRNA or CBR3-AS1 shRNA and cultured in the absence or presence of 25 mM LiCl for 8 days. Each bar represents the mean ± SD for biological triplicate experiments. 0.01 < *P < 0.05, **P < 0.01, one-way ANOVA. **Figure S4.** CBR3-AS1 regulates the LAD Wnt/β-catenin signaling. (A) Wound healing scratch assays with H1975 cells stably expressing control shRNA or CBR3-AS1 shRNA or co-transfected with FLAG-β-catenin. Each bar represents the mean ± SD for biological triplicate experiments. ^#^P > 0.05, 0.01 < *P < 0.05, **P < 0.01, one-way ANOVA. Scale bar, 50 μm. (B) H1975 cells stably expressing control shRNA or CBR3-AS1 shRNA or co-transfected with FLAG-β-catenin followed by transwell invasion assays. Each bar represents the mean ± SD for biological triplicate experiments. ^#^P > 0.05, **P < 0.01, one-way ANOVA. Scale bar, 50 μm.

**Additional file 2:** Western blotting original data.

**Additional file 3: Table S2.** Mass spectrometry analysis of lncRNA CBR3-AS1 interacting protein

**Additional file 4: Table S3.** siRNA or shRNA sequence. **Table S4.** qRT-PCR primers.

**Additional file 5: Table S1.** Patient information.

## Data Availability

All relevant data are available from the authors on request.
