# Supplementary material for: LncRNA CBR3-AS1 potentiates Wnt/β-catenin signaling to regulate lung adenocarcinoma cells proliferation, migration and invasion
Source: Cancer Cell Int. 2021 Jan 9;21:36. doi: 10.1186/s12935-020-01685-y (PMC7796595; doi:10.1186/s12935-020-01685-y)
Supplement: Supplementary file 1 — Additional file 1: Figure S1. LncRNA CBR3-AS1 is highly-expressed in LAD and cell lines. (A) The expression of lncRNA CBR3-AS1 in LAD (n = 483) or Normal (n = 347) and LSCC (n = 486) or Normal (n = 338) tissues from TCGA and GTEx database (http://gepia.cancer-pku.cn/detail.php?gene=CBR3-AS1). **P < 0.01, #P > 0.05, one-way ANOVA. Figure S2. CBR3-AS1 facilitates Wnt/β-catenin signaling activation by promoting nuclear location of β-catenin in LAD cell lines. (A) A549 cells were transfected with control siRNA or CBR3-AS1 siRNA and the expression of c-Myc, LGR5 and MMP-7 was examined by western blotting assays. Figure S3. CBR3-AS1 promotes LAD Wnt/β-catenin signaling. (A) Quantitation colony number of colony formation assays with A549 cells stably expressing control shRNA or CBR3-AS1 shRNA and cultured in the absence or presence of 25 mM LiCl for 2 weeks. Each bar represents the mean ± SD for biological triplicate experiments. **P < 0.01, one-way ANOVA. (B) Growth viability assay with H1975 cells stably expressing control shRNA or CBR3-AS1 shRNA and cultured in the absence or presence of 25 mM LiCl for 8 days. Each bar represents the mean ± SD for biological triplicate experiments. 0.01 < *P < 0.05, **P < 0.01, one-way ANOVA. Figure S4. CBR3-AS1 regulates the LAD Wnt/β-catenin signaling. (A) Wound healing scratch assays with H1975 cells stably expressing control shRNA or CBR3-AS1 shRNA or co-transfected with FLAG-β-catenin. Each bar represents the mean ± SD for biological triplicate experiments. #P > 0.05, 0.01 < *P < 0.05, **P < 0.01, one-way ANOVA. Scale bar, 50 μm. (B) H1975 cells stably expressing control shRNA or CBR3-AS1 shRNA or co-transfected with FLAG-β-catenin followed by transwell invasion assays. Each bar represents the mean ± SD for biological triplicate experiments. #P > 0.05, **P < 0.01, one-way ANOVA. Scale bar, 50 μm. [file 12935_2020_1685_MOESM1_ESM.pdf]

Additional file 1: Figure S1

A

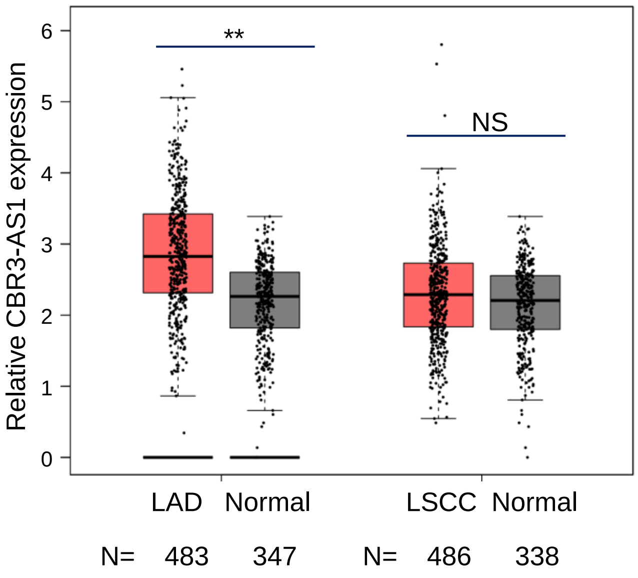

Additional file 1: Figure S2

A

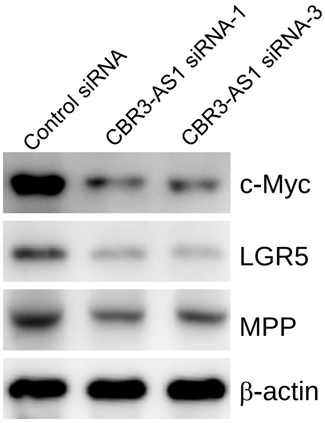

Additional file 1: Figure S3

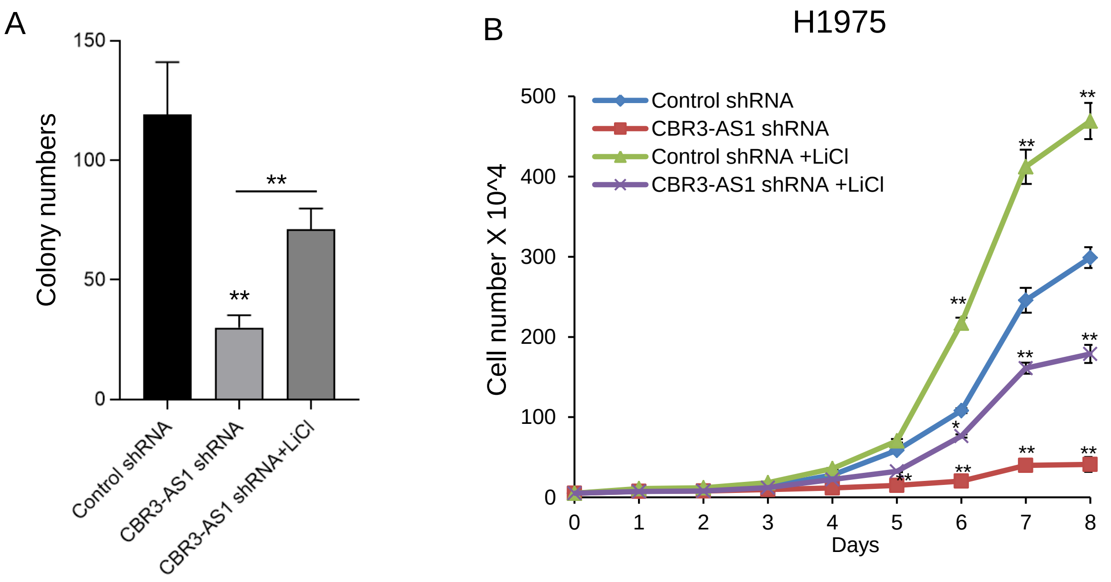

Additional file 1: Figure S4

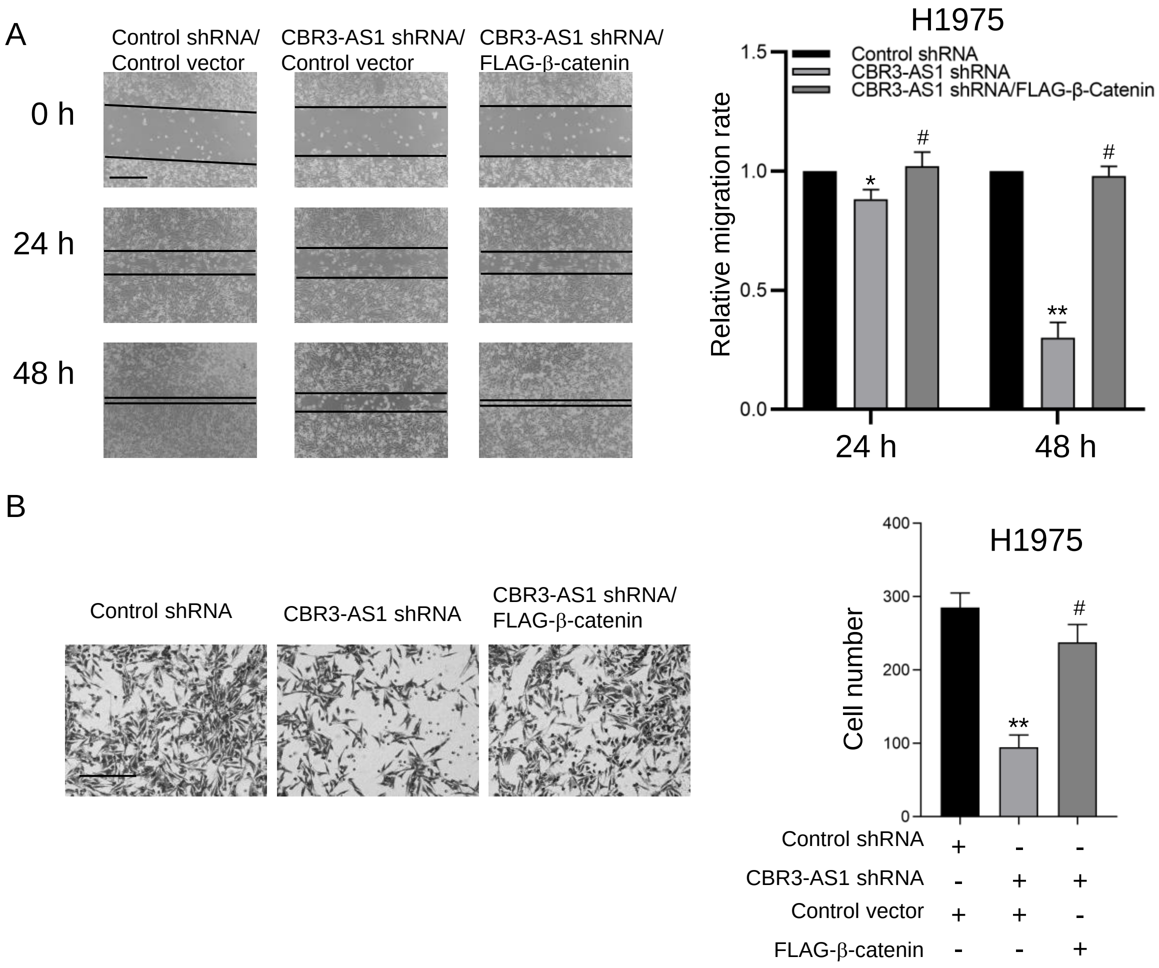

Additional file 1

Additional file 1: Figure S1. LncRNA CBR3-AS1 is highly-expressed in LAD and cell lines. (A) The expression of lncRNA CBR3-AS1 in LAD (n=483) or Normal (n=347) and LSCC (n=486) or Normal (n=338) tissues from TCGA and GTEx database (http://gepia.cancer-pku.cn/detail.php?gene=CBR3-AS1###). ***P*<0.01, $^{\#}P$>0.05, one-way ANOVA.

Additional file 1: Figure S2. CBR3-AS1 facilitates Wnt/β-catenin signaling activation by promoting nuclear location of β-catenin in LAD cell lines. (A) A549 cells were transfected with control siRNA or CBR3-AS1 siRNA and the expression of c-Myc, LGR5 and MMP-7 was examined by western blotting assays.

Additional file 1: Figure S3. CBR3-AS1 promotes LAD **cell proliferation by target Wnt/β-catenin signaling.** (A) Quantitation colony number of colony formation assays with A549 cells stably expressing control shRNA or CBR3-AS1 shRNA and cultured in the absence or presence of 25 mM LiCl for two weeks. Each bar represents the mean ± S.D. for biological triplicate experiments. **P<0.01, one-way ANOVA. (B) Growth viability assay with H1975 cells stably expressing control shRNA or CBR3-AS1 shRNA and cultured in the absence or presence of 25 mM LiCl for eight days. Each bar represents the mean ± S.D. for biological triplicate experiments. 0.01<**P*<0.05, ***P*<0.01, one-way ANOVA.

Additional file 1: Figure S4. CBR3-AS1 regulates the LAD cell migration and invasion

through Wnt/β-catenin signaling. (A) Wound healing scratch assays with H1975 cells stably expressing control shRNA or CBR3-AS1 shRNA or co-transfected with FLAG-β-catenin. Each bar represents the mean ± S.D. for biological triplicate experiments. $^{\#}P>0.05$, $0.01<{}^{*}P<0.05$, $^{**}P<0.01$, one-way ANOVA. Scale bar, 50 μm. (B) H1975 cells stably expressing control shRNA or CBR3-AS1 shRNA or co-transfected with FLAG-β-catenin followed by transwell invasion assays. Each bar represents the mean ± S.D. for biological triplicate experiments. $^{\#}P>0.05$, $^{**}P<0.01$, one-way ANOVA. Scale bar, 50 μm.
