# Supplementary material for: LncRNA CBR3-AS1 potentiates Wnt/β-catenin signaling to regulate lung adenocarcinoma cells proliferation, migration and invasion
Source: Cancer Cell Int. 2021 Jan 9;21:36. doi: 10.1186/s12935-020-01685-y (PMC7796595; doi:10.1186/s12935-020-01685-y)
Supplement: Supplementary file 2 — Additional file 2: Western blotting original data. [file 12935_2020_1685_MOESM2_ESM.pptx]

## Slide 1
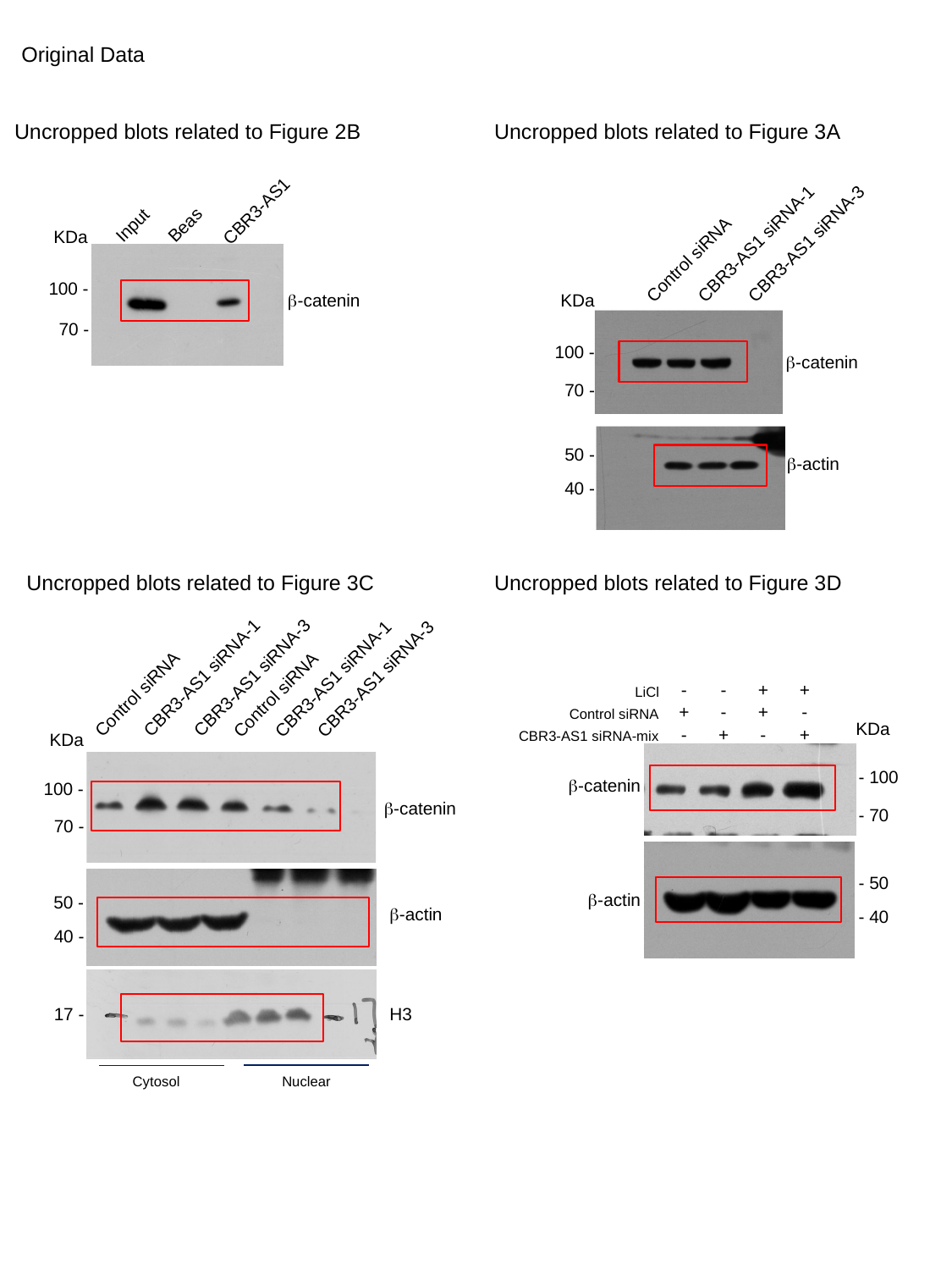

Original Data
Uncropped blots related to Figure 2B
Uncropped blots related to Figure 3A
CBR3-AS1
Beas
Input
KDa
CBR3-AS1 siRNA-1
CBR3-AS1 siRNA-3
Control siRNA
100 -
β-catenin
KDa
70 -
100 -
β-catenin
70 -
50 -
β-actin
40 -
Uncropped blots related to Figure 3C
Uncropped blots related to Figure 3D
CBR3-AS1 siRNA-1
CBR3-AS1 siRNA-3
CBR3-AS1 siRNA-1
CBR3-AS1 siRNA-3
Control siRNA
LiCl
Control siRNA
| - | - | + | + |
| --- | --- | --- | --- |
| + | - | + | - |
| - | + | - | + |
Control siRNA
KDa
CBR3-AS1 siRNA-mix
KDa
- 100
b-catenin
100 -
β-catenin
- 70
70 -
- 50
b-actin
50 -
β-actin
- 40
40 -
17 -
H3
Cytosol
Nuclear

## Slide 2
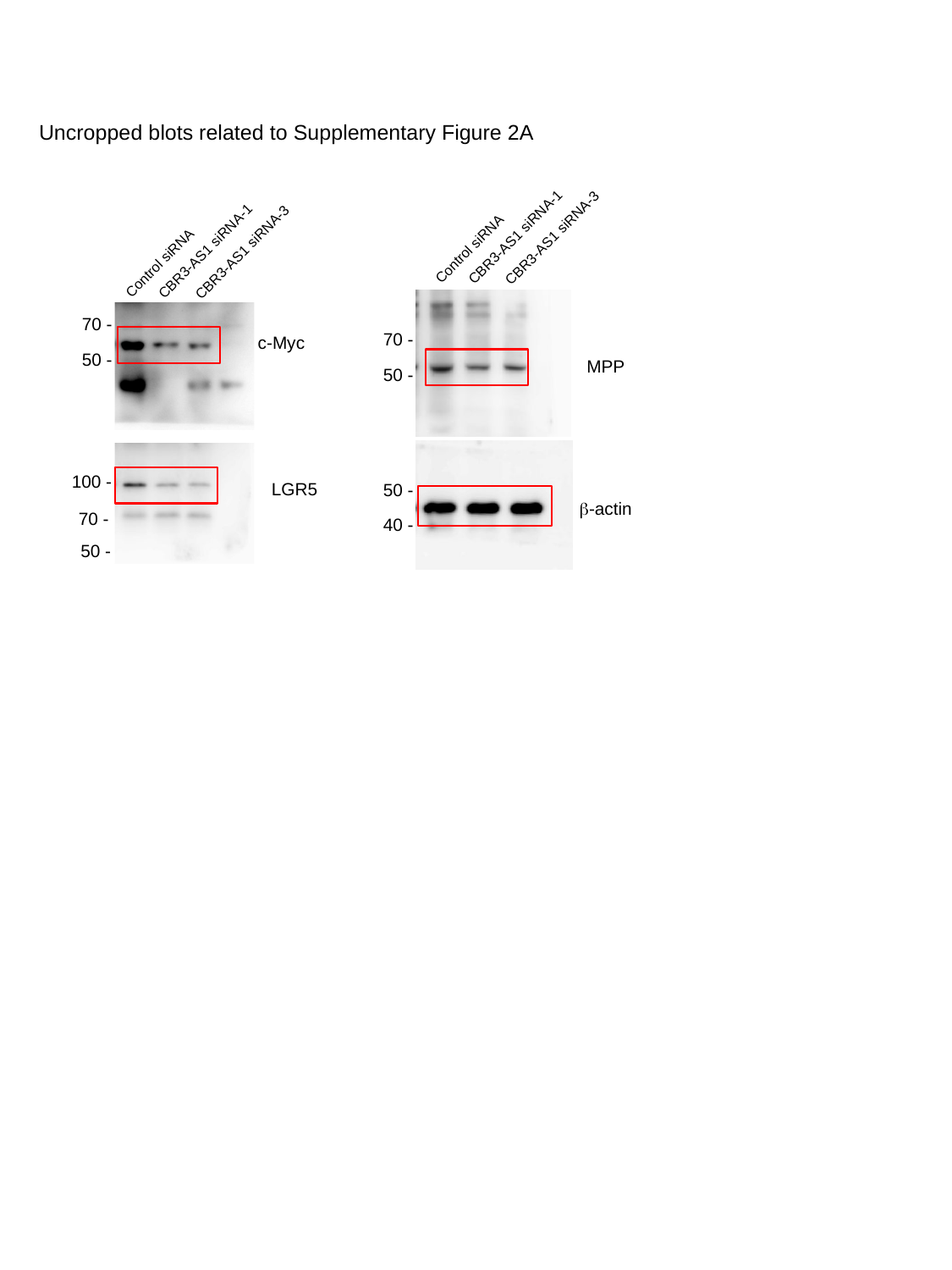

Uncropped blots related to Supplementary Figure 2A
CBR3-AS1 siRNA-1
CBR3-AS1 siRNA-3
Control siRNA
CBR3-AS1 siRNA-1
CBR3-AS1 siRNA-3
Control siRNA
70 -
70 -
c-Myc
50 -
MPP
50 -
100 -
LGR5
50 -
b-actin
70 -
40 -
50 -
