# Supplementary material for: LncRNA CBR3-AS1 potentiates Wnt/β-catenin signaling to regulate lung adenocarcinoma cells proliferation, migration and invasion
Source: Cancer Cell Int. 2021 Jan 9;21:36. doi: 10.1186/s12935-020-01685-y (PMC7796595; doi:10.1186/s12935-020-01685-y)
Supplement: Supplementary file 3 — Additional file 3: Table S2. Mass spectrometry analysis of lncRNA CBR3-AS1 interacting protein [file 12935_2020_1685_MOESM3_ESM.pdf]

Additional file 3: Table S2. Mass Spectrometry Analysis of lncRNA CBR3-AS1 interacting Protein

Molecular weights	Protein	Sequence	# PSMs	# Protein Groups	q-Value	PEP	XCorr	Charge	MH+ [Da	ΔM [ppm	RT [min]	# Missed Cleavages
85 kDa	CTNNB1											
		ITTLHNLLLHQEGAK	1	2	0	0.00001221	4.381	3	1898.94	-1.5755	26.4636	0
		mEEIVEGcTGALHILAR	1	2	0	0.0001294	3.52483	3	1914.94	-2.5723	25.4782	0
		SPQMVSAIVR	2	1	0	0.002092	3.70268	2	1405.69	-1.7377	14.6811	0
		TQFDAAHPTNVQR	1	2	0.006	0.1733	1.84281	2	881.469	-4.474	19.2473	0
		QILAYGNQESK	3	2	0	0.0002549	4.09763	2	1424.63	-1.3153	17.7924	0
		HQEAEMAQNAVR	2	1	0	0.0004343	3.42717	2	1383.64	-4.2949	14.7623	0
		AAVMVHQLSK	2	1	0	0.009959	3.33501	2	1083.6	-2.0585	15.8029	0
		TMQNTNDVETAR	3	1	0	0.0009547	3.31025	2	1379.62	-1.2644	12.8253	0
		AAVMVHQLSK	1	1	0	0.00004682	3.75644	2	1435.74	-0.5772	17.1618	0
		NEGVATYAAAVLFR	1	1	0	0.0001901	3.07608	2	1481.78	0.28532	29.8377	0
		DIGAQGEPLGYR	1	3	0	0.004656	1.81701	2	1245.64	-2.3839	24.0796	0
		TSMGGTQQQFVEGVR	1	1	0	0.006636	3.00619	2	1624.78	2.00795	21.1321	0
		QALGLHLTDPSQR	1	2	0	0.002149	2.85392	2	1350.72	-3.0233	20.0111	1
		VAAGVLcELAQDK	1	2	0	0.0002727	2.5325	2	1373.71	1.41661	24.737	0
		AGDREDITEPAIcALR	1	1	0	0.01308	2.52369	3	1786.87	-3.0663	23.4607	1
		LVQNcLWTLR	1	2	0	0.001428	2.3316	2	1302.7	-1.6692	26.4601	0
		DCLQILAYGNQESK	1	1	0.001	0.04681	1.21616	2	1053.61	-1.1613	28.7218	0
		MEEIVEGcTGALHILAR	3	2	0	0.002794	3.159	2	1221.61	-2.62	24.5724	0
		LVQLLVR	1	1	0	0.02994	1.99889	2	840.566	-1.0349	22.9754	0
		LAEPSQMLK	1	1	0	0.01766	1.75531	2	1016.55	0.59149	19.9184	0
		NLALcPANHAPLR	1	1	0	0.01702	1.54384	3	1446.76	-1.7528	20.8414	0
		GVATYAAAVLFR	1	2	0	0.001823	2.98351	2	1179.6	0.24102	20.3001	0
		LLWTTSRVLK	1	1	0	0.0153	2.21767	3	1357.72	1.08265	19.0829	1
		LLWTTSR	1	2	0	0.01816	1.2751	2	876.492	-2.2858	22.2813	0
		AIPELTK	3	2	0.001	0.05639	1.26095	2	771.461	0.37726	17.4406	0
		LAEPSQMLK	1	2	0.006	0.1733	1.84281	2	881.469	-4.474	19.2473	0
		QDDPSYR	2	2	0.006	0.1756	1.78027	2	1037.57	0.86098	18.2518	1
		CTAGTLHNLSHHR	8	4	0	0.01266	2.63142	2	1132.54	-0.7852	17.8353	0
		LLNDEDQVVVNK	3	2	0	0.002794	3.159	2	1221.61	-2.62	24.5724	0
		ADLMELDMAMEPDR	12	4	0	0.0194	2.43169	2	1106.52	-2.5462	10.9559	0
		LGKDAVEDLESVGK	2	1	0	0.0003394	3.9209	2	1459.77	1.26278	23.8167	1
		ENAGEDPGLAR	7	1	0	0.007945	2.86939	2	1128.53	-1.459	13.1851	0
		DAVEDLESVGK	4	1	0	0.002938	2.57595	2	1161.56	-2.556	23.4808	0
70 kDa	HSPA8											
		NSLESYAFNmK	1	1	0	0.002002	2.51734	2	1319.59	-2.3132	22.5088	0
		MVQEAEKYK	4	2	0	0.006154	2.17592	2	1125.56	-0.8326	13.8877	1
		iTITNDKGR	1	2	0	0.026	1.55021	2	1059.58	0.50518	17.3898	1
		FDDAVVQSDMK	3	1	0	0.00002872	4.06699	2	1410.67	-1.5653	20.1435	1
		mVQEAEKYK	1	2	0.003	0.09872	1.31159	2	1141.55	-2.7825	11.0114	1
		TFDIDANGILNVSAVDK	3	1	0	0.00003273	3.75888	2	1649.79	-1.0648	22.7	0
		EIVHIQAGQcGNQIGAK	2	1	0	0.00003157	3.65777	3	1805.9	-0.6338	21.6747	1
		MREIVHIQAGQcGNQIGAK	4	3	0	8.396E-08	6.57621	3	2110.06	-3.73	21.9661	1
		HWPFMVVNDAGR	1	1	0	0.0001322	3.51095	2	1452.68	1.71112	21.592	1
		MSMKEVDEQmLNVQNK	2	1	0	0.00119	3.48522	2	1268.66	-0.1933	20.0687	1
		MVQEAEK	3	2	0	0.0004961	3.36929	2	1659.89	-4.4004	26.734	0
		KLAVNMVPFPR	3	1	0	0.001242	3.28697	2	1303.6	-2.5904	25.4213	0
		SFYPEEVSSMVLTK	1	1	0	0.0006243	3.26524	2	1665.78	-3.6446	20.1365	0
		LHFFmPGFAPLTSR	6	1	0	0.0002632	3.19819	2	1982	0.98494	26.0629	0
		DLGGGTFDVSILTIEDGIFEVK	4	3	0	0.001565	3.19098	2	1228.63	-0.7964	15.845	0
		FPGQLNADLRK	5	2	0	0.001093	3.17372	2	1487.7	-2.031	22.977	0
		NPTNTVFDAK	3	1	0	0.001865	3.02936	2	1253.61	-2.2435	27.1398	0
		NQTAEKEEFEHQQK	3	1	0	0.003933	2.92667	2	1235.62	-3.3656	24.9907	0
		TYSCVGVFQHGK	1	1	0	0.00057	2.78539	2	1251.62	-0.7228	23.7147	0
		AILVDLEPGTMDSVR	4	1	0	0.001251	2.68372	2	1616.78	-1.9977	27.7879	0
		GDTHLGGEDFDNR	16	1	0	0.009904	2.67855	2	1180.62	-0.0815	13.598	1
51 kDa	KRT17											
		QFTSSSSIK	1	3	0.001	0.05194	1.70216	3	1965.91	-1.4715	25.3377	1
		GSSGLGGGSSR	1	1	0	0.01156	2.13691	2	1257.59	0.48907	17.6486	0
		TeVDNANILLQIDNAR	11	5	0	0.01954	2.0356	2	809.44	-0.8493	16.1757	0
		LSGGLGAGSCR	2	4	0	0.009267	1.81221	2	1148.53	-2.1925	13.695	0
		NHEEEMNALR	3	2	0	0.006735	2.93316	2	1220.6	0.37559	18.8975	0
		QALEIELQSQLSMK	3	1	0	0.008764	2.87623	2	1259.67	-0.4348	21.2348	1
		CEMEQQNQEYK	1	1	0	0.0003048	2.76762	2	2103.95	-3.4988	21.8196	0
		wTVDNANILLQIDNAR	1	1	0	0.01511	2.7659	2	1241.59	-2.2237	20.5041	0
		IGScVEEQLAQLR	8	4	0	0.01266	2.63142	2	1132.54	-0.7852	17.8353	0
		VLDELTLAR	2	3	0	0.005427	1.69751	2	1122.58	-1.6081	18.2793	0
		IELQSQLSMK	3	5	0	0.01606	1.64956	2	851.449	-2.6065	22.1001	0
		LDELTLAR	13	5	0	0.03121	1.39882	2	807.399	-0.7653	17.5905	0
		CEMEQQNQEYK	14	4	0	0.01438	2.4753	2	1064.61	-0.3444	20.452	1
		LSVEADINGLcR	12	4	0	0.0194	2.43169	2	1106.52	-2.5462	10.9559	0
		SSFGGVDGLLAGGEK	5	3	0	0.001226	2.24256	2	1029.59	-1.6809	23.4113	0
		FETEQALR	1	1	0	0.0153	2.21767	3	1357.72	1.08265	19.0829	1
		LLEGEDAHLTaQYK	2	3	0.001	0.05632	2.32957	2	1036.53	-0.5165	19.7145	1
		EaQVHQTTR	3	5	0.001	0.03324	1.96215	2	849.409	-1.2374	20.1983	0
		CEMEQQNQEYK	1	1	0.001	0.06098	1.84411	3	1897.88	2.27214	23.6706	1
		TAwTVDNANILLQIDNAR	3	2	0.002	0.07606	1.29921	2	746.403	-1.6178	14.7233	0
67 kDa	PTBP1											
		QFQALLQYADPVSAQHAK	2	2	0.006	0.1756	1.78027	2	1037.57	0.86098	18.2518	1
		DGQNIYNACCTLRIDFSK	3	1	0	0.00005086	4.68703	3	2120.04	-1.6152	20.0035	1
		VQLPREGQEDQGLTK	2	3	0	0.00005424	4.01045	2	1379.72	-4.5793	23.0898	1
		PIRITLSK	2	1	0	0.002092	3.70268	2	1405.69	-1.7377	14.6811	0
		SNIPPSVSEEDLK	5	2	0	0.007563	3.63619	2	1278.58	-1.0396	15.0564	0
		PVLRGQPIYIQFSNHK	2	1	0	0.00004617	3.48705	2	2064.14	-1.0927	27.0108	0
		DGIVPDIAVGTK	6	2	0	0.009715	3.29422	2	1106.56	-1.8951	16.0882	0
		QPIYIQFSNHK	1	1	0	0.0007037	3.2197	3	2350.09	-1.9134	18.9948	0
		FYPVTLDVLHQIFSK	6	2	0	0.001332	3.16051	2	1301.66	-2.1865	19.2705	0
		LTSLNVK	14	4	0	0.025	2.961	2	1090.53	0.25336	15.2309	0
		NHHLRVSFSK	3	1	0	0.0033	2.61256	2	1260.57	-2.3664	12.2825	0
45 kDa	ACTB											
		EYDESGPSIVHR	6	6	0	0.02836	2.28337	2	1005.46	-2.3652	13.7069	0
		VAIQAVLSLYASGR	3	5	0	0.008935	2.27951	3	1350.66	-1.5188	16.523	2
		DIAALVVDNGSGMCK	6	6	0	0.02836	2.28337	2	1005.46	-2.3652	13.7069	0
		IWHHTFYNELR	3	5	0	0.008935	2.27951	3	1350.66	-1.5188	16.523	2
		YDESGPSIVHR	1	3	0	0.004656	1.81701	2	1245.64	-2.3839	24.0796	0
		GYSFTTTAER	3	8	0	0.02081	1.5511	2	827.429	-0.7681	21.6029	0
		MATAASSSSLEK	2	2	0.001	0.03884	2.22498	3	1257.69	-2.8614	13.8568	2
		GQVITIGNER	1	6	0.002	0.08517	1.6609	2	1047.48	-0.7027	19.223	0
		ALVVDNGSGMCK	4	6	0.002	0.08188	1.60906	2	906.468	-0.4572	11.7843	0
		GYSFTTTAER	1	6	0.004	0.1362	1.04385	3	1652.91	-0.8008	28.8332	2
		HGIVTNWDDMEK	1	2	0.006	0.1733	1.84281	2	881.469	-4.474	19.2473	0
38 kDa	HNRNPA1											
		FIGGLSFETTDESLcR	1	1	0.001	0.04681	1.21616	2	1053.61	-1.1613	28.7218	0
		HFEQWGTLTDCVVMR	3	4	0.003	0.09466	1.84921	2	1065.43	-1.4589	15.44	0
		VwTYATVEEVDAAMNAR	1	3	0.003	0.1191	1.55702	2	1119.54	-0.9277	16.8818	1
		EDTEEHHLR	2	2	0	0.003042	2.69028	2	1026.52	0.27746	19.6833	0
		FAFeVTFDDHDSVDK	9	5	0	0.02494	2.68398	3	1133.56	-0.6554	14.6514	1
		YHwTVNGHNCEVR	10	3	0	0.02679	2.61383	2	1107.54	-0.9332	15.0828	0
		QEMASASSSaQR	10	3	0	0.01215	2.55195	2	1149.55	-2.975	18.0818	0
		SGSGNFGGGR	1	2	0	0.02077	2.5343	2	972.505	-6.1705	15.4848	0
		GGFGGNDNFGR	2	2	0	0.001509	2.50142	2	1407.71	-0.887	28.2992	0
		YNNaQSSNFGPMK	9	7	0	0.02363	2.36558	2	1066.52	-0.5688	12.218	1
30 kDa	HNRNPA3											
		GGLSFEwTTDDSLR	1	1	0	0.007586	3.26218	3	1783.92	-0.5127	18.5721	0
		NDFGNYSGaQQQSNYGPMK	1	1	0	0.003345	2.78091	2	1287.69	-0.3839	21.8892	0
		HTINGHNCEVK	1	1	0	0.006986	2.38218	2	1042.59	-1.4361	19.7202	0
		GGGSGNFMGcR	1	2	0	0.001618	1.88182	2	1060.52	0.156	12.2232	0
		AFeVTFDDHDTVDK	2	1	0	0.006551	1.80899	2	1358.7	-1.2675	9.96569	1
		SGGGSGGYGSR	1	2	0.001	0.04824	1.99406	2	918.503	-1.595	17.6163	0
