# Supplementary material for: LncRNA CBR3-AS1 potentiates Wnt/β-catenin signaling to regulate lung adenocarcinoma cells proliferation, migration and invasion
Source: Cancer Cell Int. 2021 Jan 9;21:36. doi: 10.1186/s12935-020-01685-y (PMC7796595; doi:10.1186/s12935-020-01685-y)
Supplement: Supplementary file 4 — Additional file 4: Table S3. siRNA or shRNA sequence. Table S4. qRT-PCR primers. [file 12935_2020_1685_MOESM4_ESM.pdf]

Additional file 4: Table S3. siRNA or shRNA sequence

siRNAs	Sequences
Control	5’-GATATGGGCTGAATACAAA-3’
CBR3-AS1-1	5’-GTCTCCTGAGCTCAGGAAA-3’
CBR3-AS1-3	5’-GTCTGGCTAGAGGTTTACC-3’
**shRNAs**	**Sequences**
Control	CCGGGATATGGGCTGAATACAAACTCGAGTTTGTATTCAGCCCATATCTTTTTG
CBR3-AS	CCGGGCCCTCATCCAGCACCCCAGTCTCGAGACTGGGGTGCTGGATGAGGGCTTTTTG
β-catenin-1	CCGG CCAGCGTTTGGCTGAACCATCCTCGAGGATGGTTCAGCCAAACGCTGGTTTTTG
β-catenin-2	CCGGGTCCGCATGGAAGAAATAGTTCTCGAGAACTATTTCTTCCATGCGGACTTTTTG

Additional file 4: Table S4. qRT-PCR Primers

Genes	Sequences
ACTB	F: CATGTACGTTGCTATCCAGGC
	R: CTCCTTAATGTCACGCACGAT
CBR3-AS1	F: CTGTCGCCCAGGCTGGAGTGC
	R: GACGCCGTGGGTCCTTCTCATC
β-catenin	F: AAAGCGGCTGTTAGTCACTGG
	R: CGAGTCATTGCATACTGTCCAT
LGR5	F: TGCTGGCTGGTGTGGATGCG
	R: GCCAGCAGGGCACAGAGCAA
MMP-7	F: GAGTGAGCTACAGTGGGAACA
	R: CTATGACGCGGGAGTTTAACAT
c-Myc	F: GGCTCCTGGCAAAAGGTCA
	R: CTGCGTAGTTGTGCTGATGT
