# Supplementary material for: LncRNA CBR3-AS1 potentiates Wnt/β-catenin signaling to regulate lung adenocarcinoma cells proliferation, migration and invasion
Source: Cancer Cell Int. 2021 Jan 9;21:36. doi: 10.1186/s12935-020-01685-y (PMC7796595; doi:10.1186/s12935-020-01685-y)
Supplement: Supplementary file 5 — Additional file 5: Table S1. Patient information. [file 12935_2020_1685_MOESM5_ESM.pdf]

Additional file 5: Table S1. Patient information

				TNM Grade		
No.	Subtype	Age	Gender	T	N	M
21033	LAD	65	F	T2	N2	M0
21036	LAD	48	M	T3	N2	M0
21040	LAD	58	F	T2	N2	M0
21043	LAD	57	F	T3	N3	M1
21046	LAD	57	M	T2	N3	M0
21049	LAD	57	F	T2	N3	M0
21053	LAD	48	M	T3	N2	M0
21054	LAD	40	M	T3	N2	M1
21055	LAD	38	M	T2	N2	M0
21059	LAD	51	M	T2	N2	M0
21062	LAD	68	M	T3	N2	M0
21064	LAD	55	M	T2	N3	M0
21065	LAD	57	F	T2	N2	M0
21067	LAD	43	M	T2	N3	M0
21069	LAD	31	M	T2	N3	M0
21071	LAD	45	F	T2	N2	M1
21074	LAD	66	M	T3	N2	M1
21076	LAD	58	F	T2	N2	M0
21077	LAD	57	M	T2	N2	M0
21081	LAD	59	M	T2	N2	M0
21084	LAD	43	F	T2	N3	M0
21085	LAD	48	M	T2	N2	M0
21087	LAD	40	F	T3	N3	M1
21090	LAD	45	M	T2	N3	M0
21093	LAD	66	M	T2	N2	M1
21094	LAD	68	M	T3	N2	M0
21095	LAD	55	M	T3	N2	M1
21098	LAD	55	M	T2	N2	M0
21101	LAD	57	M	T2	N2	M0
21104	LAD	43	M	T3	N2	M0
21106	LAD	65	M	T2	N2	M0
21107	LAD	45	F	T3	N3	M1
21108	LAD	48	M	T2	N3	M0
21111	LAD	58	F	T2	N3	M0
21113	LAD	57	F	T3	N2	M0
21115	LAD	59	M	T3	N2	M1
21116	LAD	57	F	T2	N2	M0
21119	LAD	58	F	T2	N2	M0
21123	LAD	49	F	T3	N2	M0
21124	LAD	41	M	T2	N3	M0
21127	LAD	39	F	T2	N2	M0
22630	LAD	52	M	T2	N3	M0
22632	LAD	56	M	T2	N3	M0
22635	LAD	70	M	T2	N2	M1
22638	LAD	32	M	T3	N2	M1
22640	LAD	66	M	T2	N2	M0
22641	LAD	46	M	T2	N2	M0
22644	LAD	67	M	T2	N2	M0
22647	LAD	58	M	T2	N3	M0
22649	LAD	58	M	T2	N2	M0
22650	LAD	60	M	T3	N3	M1

22650	LAD	44	M	T2	N3	M0
22653	LAD	41	M	T2	N2	M1
22654	LAD	39	F	T3	N2	M0
22657	LAD	46	M	T3	N2	M1
22663	LAD	67	M	T2	N2	M0
22664	LAD	56	M	T2	N2	M0
22666	LAD	70	F	T3	N2	M0
22668	LAD	32	M	T2	N2	M0
22670	LAD	66	M	T3	N3	M1
22666	LSC	46	M	T2	N3	M0
22668	LSC	67	F	T2	N3	M0
22670	LSC	58	F	T3	N2	M0
22671	LSC	58	M	T3	N2	M1
22673	LSC	60	F	T2	N2	M0
22675	LSC	44	M	T2	N2	M0
22678	LSC	41	F	T3	N2	M0
22678	LSC	39	M	T2	N3	M0
22680	LSC	46	F	T2	N2	M0
22620	LSC	67	F	T2	N3	M0
22621	LSC	69	M	T2	N3	M0
22624	LSC	58	F	T2	N2	M1
22626	LSC	58	M	T3	N2	M1
22628	LSC	44	M	T2	N2	M0
22629	LSC	49	F	T2	N2	M0
22632	LSC	39	M	T2	N2	M0
22635	LSC	52	M	T2	N3	M0
22636	LSC	67	M	T2	N2	M0
22638	LSC	69	F	T3	N3	M1
22641	LSC	58	M	T2	N3	M0
22644	LSC	59	M	T2	N2	M1
22646	LSC	59	M	T3	N2	M0
22647	LSC	61	M	T3	N2	M1
22650	LSC	45	F	T2	N2	M0
22652	LSC	50	F	T2	N2	M0
22654	LSC	40	F	T3	N2	M0
22657	LSC	53	F	T2	N2	M0
22660	LSC	68	F	T3	N3	M1
22661	LSC	70	M	T2	N3	M0
22664	LSC	62	M	T2	N3	M0
22666	LSC	48	M	T3	N2	M0
22667	LSC	36	M	T3	N2	M1
22668	LSC	70	F	T2	N2	M0
22671	LSC	62	F	T2	N2	M0
22674	LSC	64	M	T3	N2	M0
22675	LSC	62	M	T2	N3	M0
22676	LSC	48	F	T2	N2	M0
22643	LSC	45	M	T2	N3	M0
22649	LSC	43	M	T2	N3	M0
22661	LSC	56	M	T2	N2	M1
24191	LSCC	60	F	T2	N2	M0
24192	LSCC	62	F	T2	N2	M0
21038	LSCC	66	M	T2	N3	M0
21050	LSCC	43	M	T2	N2	M1
21061	LSCC	66	F	T2	N2	M0

21073	LSCC	48	M	T3	N2	M0
21082	LSCC	57	M	T3	N2	M0
21091	LSCC	51	M	T2	N3	M0
21105	LSCC	31	F	T2	N3	M0
21112	LSCC	57	M	T2	N2	M1
21121	LSCC	44	M	T2	N2	M0
22636	LSCC	44	F	T3	N2	M0
22646	LSCC	59	M	T3	N2	M0
22651	LSCC	49	M	T2	N3	M0
22667	LSCC	44	M	T2	N3	M0
22669	LSCC	59	M	T2	N2	M1
22677	LSCC	49	M	T2	N2	M0
22625	LSCC	60	F	T3	N2	M0
22633	LSCC	46	M	T3	N2	M0
22643	LSCC	70	F	T2	N3	M0
22656	LSCC	47	M	T2	N3	M0
22665	LSCC	74	M	T2	N2	M1
22672	LSCC	62	M	T2	N2	M0
22689	LSCC	71	F	T3	N2	M0
21034	LSCC	45	M	T2	N2	M0
21047	LSCC	59	M	T2	N3	M0
21057	LSCC	45	M	T2	N2	M0
21066	LSCC	69	M	T3	N3	M1
21070	LSCC	65	M	T2	N3	M0
21079	LSCC	57	F	T2	N2	M0
21088	LSCC	38	M	T2	N3	M0
21097	LSCC	57	F	T2	N2	M0
21102	LSCC	69	F	T2	N2	M0
21109	LSCC	66	M	T2	N3	M0
21117	LSCC	60	F	T2	N2	M0
21130	LSCC	46	M	T3	N3	M1
22633	LSCC	58	M	T2	N3	M0
22643	LSCC	49	F	T2	N2	M0
22649	LSCC	58	M	T2	N3	M0
22661	LSCC	52	F	T2	N2	M0
